# Therapeutic evaluation of microRNA-15a and microRNA-16 in ovarian cancer

**DOI:** 10.18632/oncotarget.7618

**Published:** 2016-02-23

**Authors:** Shailendra Kumar Dhar Dwivedi, Soumyajit Banerjee Mustafi, Lingegowda S. Mangala, Dahai Jiang, Sunila Pradeep, Cristian Rodriguez-Aguayo, Hui Ling, Cristina Ivan, Priyabrata Mukherjee, George A. Calin, Gabriel Lopez-Berestein, Anil K. Sood, Resham Bhattacharya

**Affiliations:** ^1^ Department of Obstetrics and Gynecology, Stephenson Cancer Center, University of Oklahoma Health Science Center, Oklahoma City, OK, USA; ^2^ Department of Cell Biology, University of Oklahoma College of Medicine, Oklahoma City, OK, USA; ^3^ Department of Pathology, Stephenson Cancer Center, University of Oklahoma Health Science Center, Oklahoma City, OK, USA; ^4^ Department of Gynecologic Oncology, The University of Texas MD Anderson Cancer Center, Houston, TX, USA; ^5^ Department of Cancer Biology, The University of Texas MD Anderson Cancer Center, Houston, TX, USA; ^6^ The Center for RNA Interference and Non-Coding RNA, The University of Texas MD Anderson Cancer Center, Houston, TX, USA; ^7^ Department of Experimental Therapeutics, The University of Texas MD Anderson Cancer Center, Houston, TX, USA

**Keywords:** BMI1, microRNA, ovarian cancer, EMT, cisplatin sensitivity

## Abstract

Treatment of chemo-resistant ovarian cancer (OvCa) remains clinically challenging and there is a pressing need to identify novel therapeutic strategies. Here we report that multiple mechanisms that promote OvCa progression and chemo-resistance could be inhibited by ectopic expression of miR-15a and miR-16. Significant correlations between low expression of miR-16, high expression of BMI1 and shortened overall survival (OS) were noted in high grade serous (HGS) OvCa patients upon analysis of The Cancer Genome Atlas (TCGA). Targeting BMI1, *in vitro* with either microRNA reduced clonal growth of OvCa cells. Additionally, epithelial to mesenchymal transition (EMT) as well as expression of the cisplatin transporter ATP7B were inhibited by miR-15a and miR-16 resulting in decreased degradation of the extra-cellular matrix and enhanced sensitization of OvCa cells to cisplatin. Nanoliposomal delivery of the miR-15a and miR-16 combination, in a pre-clinical chemo-resistant orthotopic mouse model of OvCa, demonstrated striking reduction in tumor burden compared to cisplatin alone. Thus, with the advent of miR replacement therapy some of which are in Phase 2 clinical trials, miR-15a and miR-16 represent novel ammunition in the anti-OvCa arsenal.

## INTRODUCTION

Recent reports have underscored the importance of microRNA (miR) regulatory networks in the pathogenesis of ovarian cancer (OvCa), regulating epithelial to mesenchymal transition (EMT) [1–3] and chemo-resistance [4, 5]. We previously reported that BMI1(B lymphoma Mo-MLV insertion region 1) is a direct target of miR-15a and miR-16 and their expression levels correlated inversely with the protein levels of BMI1 both in high grade serous (HGS) OvCa patient samples and cell lines [6]. Furthermore the miR-15a/16−1 locus is lost in 23.9% of ovarian and 24.7% of breast cancers [7] and deletion or downregulation of these miRs accelerates cell division by modulating the expression of genes involved in cell cycle progression [8].

OvCa is the leading cause of morbidity among gynecologic malignancies in the USA [9] and is often diagnosed only at an advanced stage. The majority of OvCa patients respond to initial therapy with tumor cytoreductive surgery and platinum/taxane-based chemotherapy [10]. However, approximately 70% of advanced stage patients develop recurrent cancer and eventually succumb to recurrent disease typically characterized by multiple drug resistance [11]. Therefore, clearly there is a compelling need for improvement in existing therapy.

BMI1, a member of the Polycomb Repressor Complex 1 (PRC1) that mediates gene repression by regulating chromatin structure, is preferentially expressed in stem cells where it supports self-renewal and clonal growth such as in glioblastoma and colorectal cancer [12–14]. Importantly, BMI1 is frequently upregulated and its expression correlates with poor prognosis and therapy failure in several types of cancer including OvCa [15–18] Experimental reduction of BMI1 increases susceptibility to cytotoxic agents and radiation therapy [15, 19]. Thus BMI1 has emerged as an important therapeutic target in several different malignancies.

Here, we investigated whether targeting BMI1 by miR-15a, miR-16 or their combination would be efficacious in inhibiting growth of chemo-resistant OvCa. While data from The Cancer Genome Atlas (TCGA) supported correlations between low miR-16 expression, high BMI1 expression and shortened overall survival (OS), *in vitro* targeting with these microRNAs a) decreased growth rate; b) decreased anchorage independent clonal growth; c) enhanced sensitivity to cisplatin; d) decreased expression of the drug efflux transporter ATP7B; e) inhibited EMT and f) decreased degradation of the extra-cellular matrix (ECM) by OvCa cells. A striking decrease in tumor growth was observed in the chemo-resistant orthotopic OvCa mouse model upon *in vivo* nanoliposomal delivery of miR-15a, miR-16 or their combination. Importantly the combination therapy of miR-15a and miR-16 without cisplatin demonstrated a significantly better response compared to either cisplatin alone or any single microRNA in combination with cisplatin. With the advent of miRNA replacement therapy some of which are in Phase 2 clinical trials [20], our results purport possible application of miR-15a and miR-16 therapy in OvCa.

## RESULTS

### Expression of miR-15a and miR-16 in ovarian cancer

**Figure 1:**
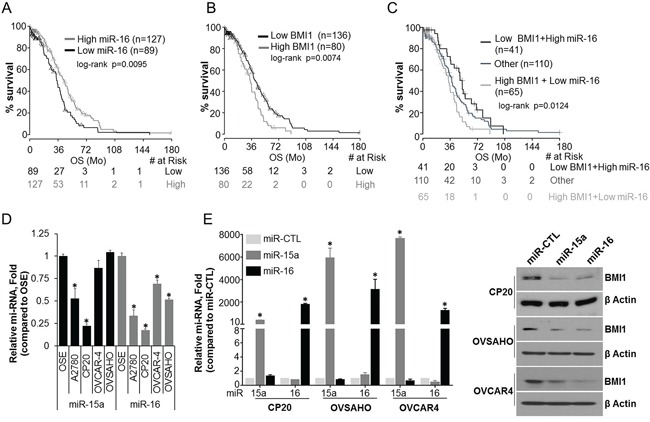
Expression and pathological significance of miR-15a, miR-16 and BMI1 in OvCa **A.** Kaplan-Meier overall survival curves in miR-16 low and high expression OvCa cases from TCGA. The percent probability of survival is plotted versus time since diagnosis in months. **B.** Kaplan-Meier overall survival curves in BMI1 low and high expression OvCa cases from TCGA. The percent probability of survival is plotted versus time since diagnosis in months. **C.** Kaplan-Meier overall survival curves for OvCa patients corresponding to low BMI1/high miR-16, high BMI1/low miR-16 and the rest of the cases (Other; using fixed cut-off for the BMI1 and miR-16 expression). **D.** Total RNA was collected using the TRI Pure reagent and subjected to RT-qPCR. The comparative C_t_ method was used to calculate the relative abundance of miR-15a or miR-16 with respect to U6 expression. Data represented as mean relative fold difference ± SD with respect to OSE cells *, P<0.05. **E.** CP20, OVSAHO and OVCAR4 cells were transfected with miR CTL, miR-15a or miR-16. After 48h of transfection microRNA levels were determined as in D (Data represent mean ± SD) and total cellular lysates were immunoblotted for BMI1 and β-actin.

Previous studies have shown that BMI1 is overexpressed in OvCa [6, 21, 22] and is a direct target of miR-15a [6] and miR-16 [6, 23]. Here we first probed the TCGA data base to evaluate the correlation between miR-15a or miR-16 expression levels and OS in high grade-serous OvCa patient samples. While a significant association between shortened OS and lower expression of miR-16 was observed (median OS, 34 months in low miR-16 vs 43.3 months in high miR-16) (Figure 1A), miR-15a did not reach statistical significance (data not shown). Expression levels of BMI1, the target of these microRNAs also significantly correlated with poor OS (median OS, 34 months in high BMI1 vs 41.4 months in low BMI1) (Figure 1B). Importantly, OS was significantly shorter in the combined BMI1 high, miR-16 low group (median OS 34 months) versus the BMI1 low, miR-16 high group (median OS 51.9 months) (Figure 1C) indicative of a direct involvement of miR-16 and BMI1 in OvCa progression and pathogenesis. We next evaluated the expression of miR-15a and miR-16 in the immortalized ovarian surface epithelial cells (OSE), A2780, cisplatin resistant CP20 [24] and high grade serous OVCAR4 [25] and OVSAHO [25] cell lines by quantitative real-time PCR (RT-qPCR). While expression of miR-16 was significantly lower in all the cell lines compared to OSE, the expression of miR-15a was significantly lower in A2780 and CP20 (Figure 1D). Efficient transfection of miR-15a or miR-16 was confirmed by RT-qPCR and they significantly decreased the expression of BMI1 in CP20, OVCAR4 and OVSAHO cells respectively (Figure 1E). This is in line with previous publications [6, 23] and supports a correlation between microRNA expression and BMI1 expression that relates to overall patient survival.

### miR-15a and miR-16 affect clonal growth and chemosensitivity

**Figure 2:**
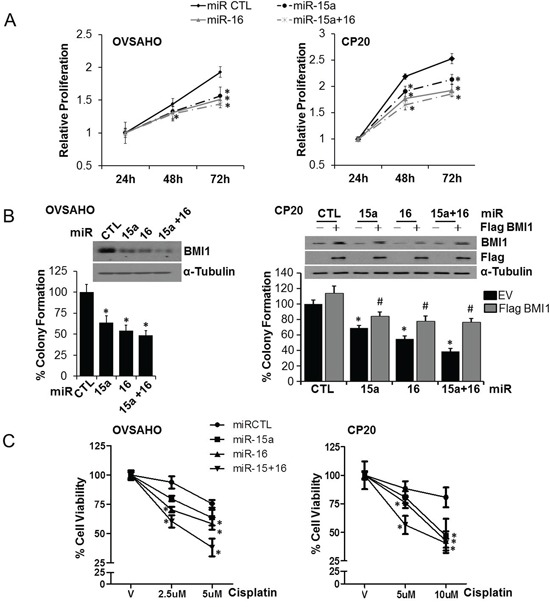
Effect of miR-15a and miR-16 on proliferation, clonal growth and chemosensitivity in OvCa **A.** OVSAHO or CP20 cells were transfected with miR-CTL, miR-15a, miR-16 or miR-15a+ miR-16. 24h after transfection, cells were counted and re-plated in 96-well plate and relative proliferation was evaluated using the CyQUANT® NF Cell Proliferation Assay Kit at indicated time points **B.** OVSAHO and CP20 cells were transfected with respective microRNAs, additionally CP20 cells were also transfected with the Flag-BMI1 construct that is non-responsive to the microRNAs. 48 h after transfection cells were re-plated in 0.3% agar and remaining cells subjected to immunoblotting for BMI1 and Flag, shown in the inset. After 10 (CP20) or 14 (OVSAHO) days, colonies were stained with crystal violet, imaged and 9 images from 3 independent experiments were quantified using ImageJ in a blinded fashion. **C.** OVSAHO and CP20 cells were transfected with miR CTL, miR-15a, miR-16 or miR-15a+ miR-16 for 24h and cells re-plated in 96-well plates. Cells were treated with cisplatin for 48h at the indicated concentrations and viability was determined using CyQUANT® NF Cell Proliferation Assay. Percent cell viability was evaluated by comparing with respective vehicle (V) treated cells. Data represent mean ± SD. *, P<0.05.

We evaluated the effect of miR-15a and/or miR-16 on growth rate of OVSAHO and CP20 cells using the fluorescence based CyQUANT® NF Cell Proliferation Assay and a moderate but consistent reduction in proliferation rate was observed in both the cell lines compared to the non-target miR control (miR CTL) (Figure 2A). The anchorage independent clonal growth of cancer cells in semi-solid medium reflects the potency of tumor cells to survive and grow in secondary locations *in vivo* and correlates closely with tumorigenicity in animal models [26]. Thus, we evaluated the effect of ectopic miR-15a or miR-16 expression on anchorage independent clonal growth in OVSAHO and CP20 cells. Significant reduction in BMI1 levels upon transfection with respective microRNAs confirmed targeting (Figure 2B). Compared to the control, significant reduction in number of colonies in miR-15a (decreased by ∼36% in OVSAHO and ∼35% in CP20), miR-16 (decreased by ∼45% in OVSAHO and ∼47% in CP20) or the miR-15a + miR-16 (decreased by ∼55% in OVSAHO and ∼63% in CP20) transfected cells were observed (Figure 2B). To establish that the decrease in colony number is mediated through BMI1, CP20 cells were co-transfected with miR-15a or miR-16 along with a Flag-BMI1 construct, non-responsive to these microRNAs. Expression of BMI1 significantly rescued the anchorage independent growth of CP20 cells (∼15% in miR-15a, ∼23% in miR-16 and ∼38% in miR-15a+miR-16) (Figure 2B). Having established that miR-15a and miR-16 could reduce growth rate and anchorage independent clonal growth we next evaluated the effect of ectopic miR expression on cisplatin sensitivity using the CyQUANT® NF Cell Proliferation Assay in OVSAHO and CP20 cells. In OVSAHO control-miR transfected cells, 5μM cisplatin for 48h decreased viability by ∼25% while miR-15a, miR-16 and miR-15a + miR-16 resulted in ∼36%, ∼40% and ∼62% reduced viability respectively (Figure 2C). In the cisplatin resistant CP20 cells, 5μm cisplatin reduced viability by ∼12% and ∼19% in control-miR and miR-15a transfected cells, while transfection with miR-16 and mir15a +16 significantly reduced the viability by ∼24% and ∼43% respectively (Figure 2C). Thus ectopic expression of miR-15a, miR-16 or their combination reduces cell proliferation rate and anchorage-independent clonal growth and increases *in vitro* cisplatin sensitivity in OvCa cells.

### miR-15a and miR-16 affect drug efflux transporter, ATP7B and EMT

**Figure 3:**
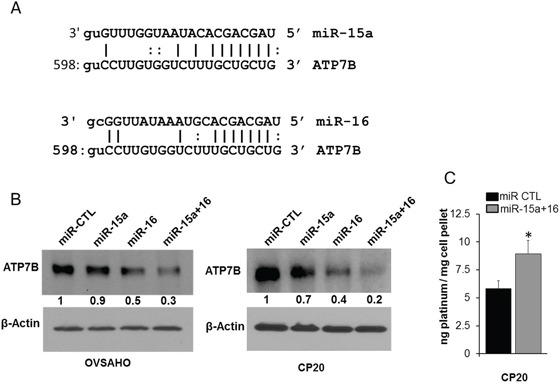
Effect of miR-15a and miR-16 on ATP7B and intracellular cisplatin **A.** Putative miR-15a and miR-16 interaction sites on ATP7B (NM_00053;NM_001005918) is represented. **B.** miR CTL, miR-15a, miR-16 or miR-15a+ miR-16 transfected OVSAHO and CP20 cells were immunobloted for ATP7B. Numbers bellow each panel indicates band intensity quantified using Image J. Values were first normalized with respect to β-Actin and then respective miR-CTL was set to 1. **C.** Intracellular platinum concentration was measured using ICP-MS in miR CTL or miR-15a+miR-16 transfected CP20 cells treated with cisplatin for 48h and mean ± SD is plotted. *, P<0.05.

In OvCa increased expression of the drug efflux transporters correlates with chemo-resistance and silencing the copper transporter ATP7B enhances cisplatin sensitivity [27]. Therefore miR prediction databases were probed for possible drug efflux transporters that are targeted by miR-15a or miR-16. Interestingly Targetscan [28], Miranda [29], microtT [30], PITA [31] all predicted ATP7B to be a putative target of miR-15a and miR-16 (Figure 3A). Transfection with miR-15a, miR-16 or their combination reduced expression of ATP7B in both OVSAHO and CP 20 cells compared to the control (Figure 3B). To determine the effect of decreased ATP7B in intracellular cisplatin accumulation, cisplatin resistant CP20 cells transfected with miR control or the combination of miR-15a+miR-16, treated with 10 μM cisplatin for 48h and intracellular platinum measured using ICP-MS [32]. Compared to the control, intracellular platinum concentration was significantly higher in the miR-15a+miR-16 treated group (miR CTL group, 5.8 and miR-15a+miR-16 group, 8.9 ng platinum/mg cell pellet) (Figure 3C). These results are significant in that standard chemotherapy for OvCa involves treatment with a platinum/taxane regimen and ATP7B is the efflux transporter for cisplatin which is affected by miR-15a and miR-16.

**Figure 4:**
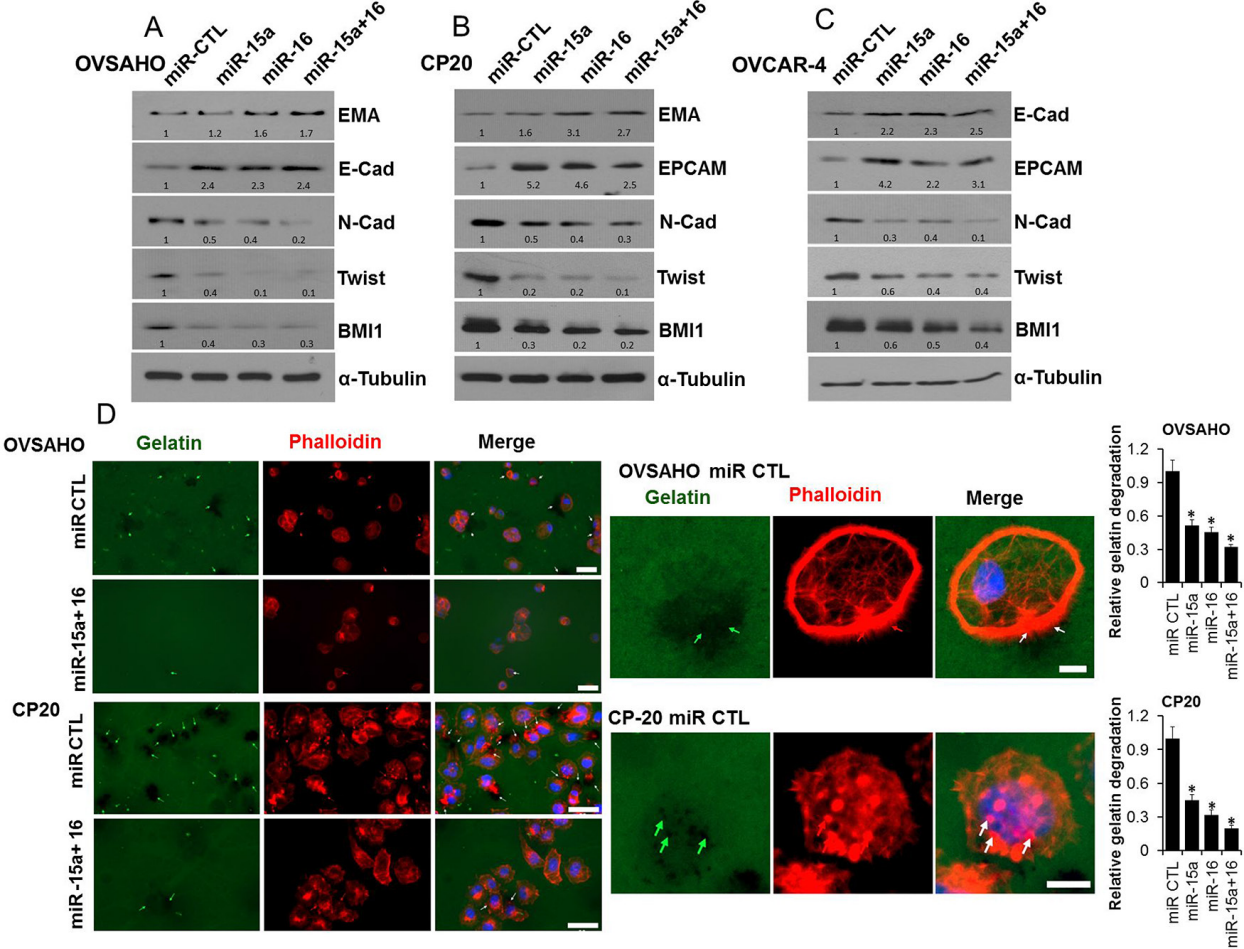
Effect of miR-15a and miR-16 on EMT and ECM degradation **A.** OVSAHO, **B.** CP20 and **C.** OVCAR4 cell lines were transfected with miR CTL, miR-15a, miR-16 or miR-15a+ miR-16 for 48h and immunobloted for respective EMT markers as indicated and BMI1. Numbers at the bottom of each panel indicate band intensity quantified using Image J. Values were first normalized with respect to α-Tubulin and then respective miR-CTL was set to 1. **D.** miR transfected OVSAHO and CP20 cells were plated on Oregon Green® 488 Gelatin coated coverslips for 48h, fixed, stained with Alexa Fluor® 555 Phalloidin, mounted in Vectashield mounting medium containing DAPI and images were acquired. At least 100 cells from three independent experiments were quantified. Scale bar on the left panel represent 40μm and on the right panel represent 10μm. Arrows indicate areas of gelatin degradation that is quantified and presented as mean ±SD relative to miR control cells. Complete panel including images of miR-15a and miR-16 are provided as Supplementary Figure 1. *, P<0.05.

Emerging evidence suggests that acquisition of invasiveness and resistance to chemotherapeutics in OvCa cells is accompanied by the loss of epithelial features and the gain of a mesenchymal phenotype, a process known as epithelial-to-mesenchymal transition (EMT) [1, 33]. Therefore we determined the expression of epithelial and mesenchymal markers after transfection with respective microRNAs in OvCa cells. In OVSAHO the epithelial marker epithelial membrane antigen (EMA) significantly increased in the miR-16 and the miR-15a+miR-16 transfected cells while E Cadherin (E-Cad) increased with all the miR combinations compared to the control (Figure 4A). The mesenchymal markers N Cadherin (N-Cad), Twist1 and as expected BMI1, all significantly decreased with all the miR combinations compared to the control (Figure 4A). Like OVSAHO, in CP20 expression of EMA increased in the miR-16 and the miR-15a+miR-16 transfected cells while E-Cad could not be detected; however expression of the epithelial cell adhesion molecule (EPCAM) increased with all the miR combinations compared to the control (Figure 4B). Again the mesenchymal markers Twist1, N-Cad and BMI1 were decreased with all the miR combinations compared to the control (Figure 4B). A similar increase in expression of epithelial markers and decrease in expression of mesenchymal markers was observed in the OVCAR4 cells (Figure 4C). To further evaluate the effect of miRs on the invasive potential, the Gelatin matrix degradation assay was utilized. Tumor cells degrade the extra-cellular matrix through invasive protrusions that are sites for targeted secretion of metalloproteases and the Gelatin matrix degradation assay allows this quantitation [34, 35]. In both OVSAHO and CP20 all the miR combinations significantly decreased the number of cells competent to degrade matrix (Figure 4D and Supplementary Figure 1). Thus in addition to affecting EMT miR-15a, miR-16 or their combination decrease the ECM degradative capacity of OvCa cells.

### Efficacy of miR-15a or miR-16 therapy in an orthotopic chemo-resistant mouse model

**Figure 5:**
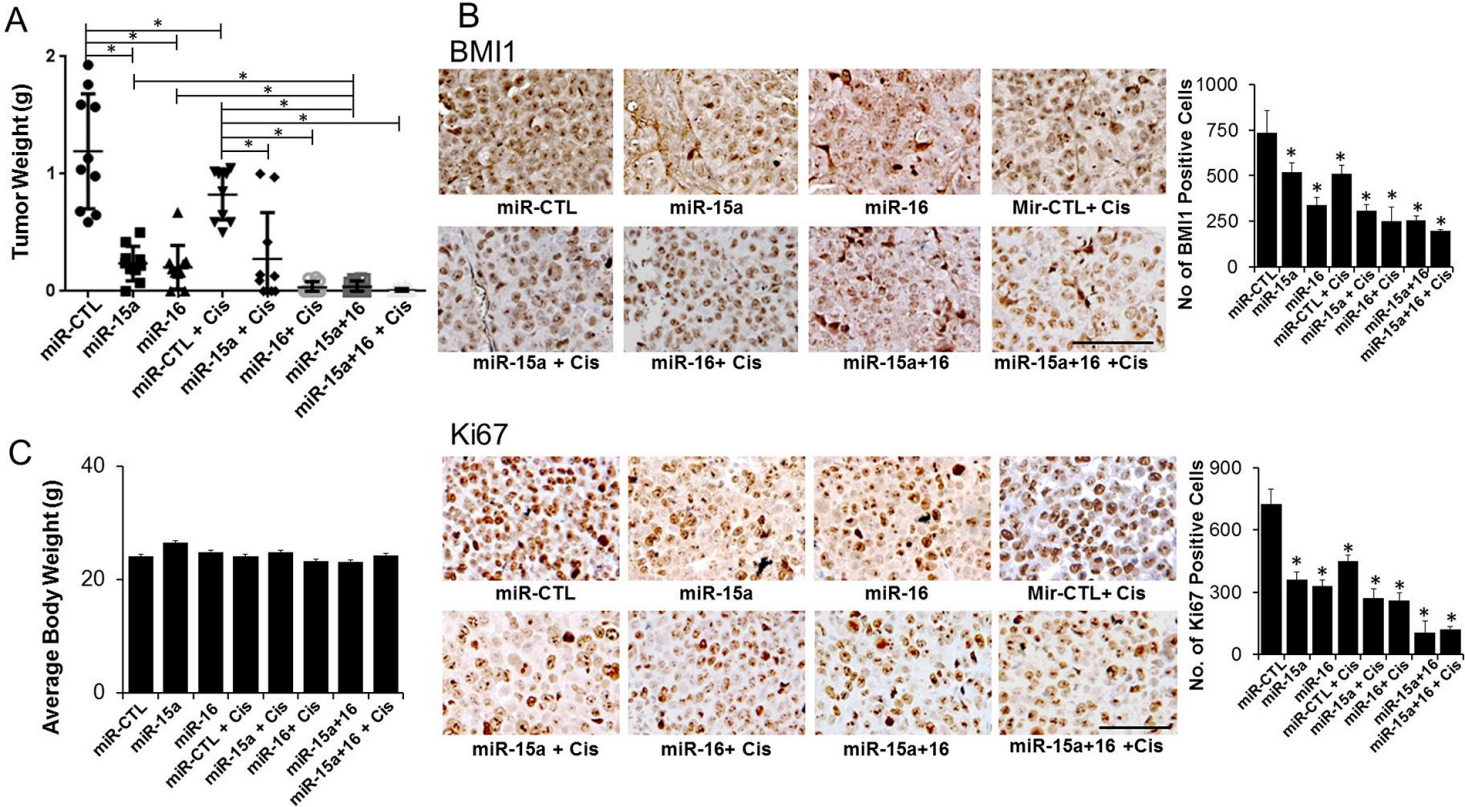
Therapeutic efficacy of miR-15a and miR-16 in the chemo-resistant orthotopic mouse model To assess the effects of miR therapy on tumor growth, treatment was initiated 1 week after i.p. injection (1×10^6^ CP20) of tumor cells. Mice were divided into eight groups (n=10), treated and sacrificed after 4 weeks of tumor cell injection. **A.** Average tumor weight ± SD from the different treatment groups are plotted **B.** Immunohistochemical staining of tumors from mice xenografts for BMI1 (upper panel) and Ki67 (lower panel). Original magnification 100X, quantification is shown graphically on the right, scale bar 100 μM. *, P<0.05. **C.** Average body weight of mice from each experimental group before sacrifice is represented.

We next evaluated the therapeutic efficacy of miR-15a and miR-16 in the orthotopic CP20 mouse model using the DOPC nanoliposomal delivery platform [1]. To simulate the treatment of advanced small-volume disease, therapy was initiated 1 week after tumor cell injection [15]. Treatment with miR-15a and miR-16 alone resulted in significant reduction in tumor weight (73% and 81%) compared to the control miR treated mice (Figure 5A). Importantly, the combination therapy of miR-15a and miR-16 without cisplatin demonstrated a better response (∼94%) compared to cisplatin alone or either single microRNAs in combination with cisplatin (Figure 5A). Compared to the control, significant decrease in the expression of BMI1 was confirmed by immunohistochemistry (IHC) in miR-15a, miR-16 or the combination treated groups (Figure 5B). Furthermore IHC for Ki67 demonstrated a significant decrease in *in vivo* tumor cell proliferation in all the treatment groups compared to the control (Figure 5B). No obvious toxicity was noted in the animals during the experiment, as assessed by changes in behavior, feeding habits, and mobility with the mean body weight remaining similar between the treatment groups (Figure 5C). These results show that delivering the combination of miR-15a and miR-16 is more efficacious than delivering either single microRNA with cisplatin.

## DISCUSSION

Here, we evaluated the efficacy of miR-15a and miR-16 in inhibiting the growth of chemo-resistant OvCa. We report that multiple mechanisms that promote OvCa progression and chemo-resistance such as clonal growth, EMT and the cisplatin efflux pump, ATP7B are inhibited by miR-15a and miR-16 resulting in a striking reduction in tumor growth in a pre-clinical chemo-resistant orthotopic mouse model of OvCa.

The clinical relevance of low miR-16 or high BMI1 expression in HGS OvCa is exemplified by the fact that these patients had significantly shortened OS. Though expression of miR-15a did not show a significant correlation with OS, we previously showed that in 28 out of 38 HGS OvCa tissues, low expression of miR-15a correlated with high BMI1 protein levels [6]. Subsequently several studies have reported significant downregulation of miR-15a and miR-16 in ovarian tumors, and these were associated with genomic copy number loss or epigenetic silencing or were due to compromised miR processing through reduced expression of Dicer [7, 36, 37]. In accordance with previous reports, expression of miR-15a or miR-16 potently downregulated BMI1 protein levels in OvCa cells [6, 23]. Expression of miR-15a or miR-16 in OvCa also decreased anchorage independent clonal growth that could be significantly but not completely rescued by Flag-BMI1 thereby suggesting existence of other biologically relevant targets of miR-15a or miR-16.

From a therapy perspective, we asked if treatment with these microRNAs would affect cisplatin sensitivity and if so how. The combination of miR-15a and miR-16 demonstrated striking sensitivity to cisplatin compared to control, we believe partly due to enhanced cisplatin accumulation in the cells facilitated by inhibition of ATP7B as has been reported previously [27]. We also confirm that ATP7B is a likely target of miR-15a and miR-16 as evidenced by decreased protein levels in miR transfected OvCa cells, however further experimental verification is required. Though acquired resistance to cisplatin is multifactorial in origin; however, impaired accumulation of drug is the single most commonly observed alteration when cells selected for resistance are compared with their sensitive equivalents [38, 39]. In corroboration, a direct relationship between the copper efflux transporter ATP7B and resistance to cisplatin has been described in several studies [38, 40–42]. Additionally, cumulating evidences suggest that acquisition of invasiveness and chemo-resistance in OvCa is a function of enhanced EMT [1, 3, 43–45]. In this context our data demonstrating inhibition of EMT and decreased ECM degradative capacity of miR-15a and mir-16 treated cells indicate the ability of these microRNAs to inhibit multiple pathways involved in OvCa tumor progression and chemo-resistance. In corroboration, a striking ∼94% inhibition in tumor growth in the miR-15a/miR-16 combination group was observed compared to cisplatin alone. We previously demonstrated that siRNA mediated targeting of BMI1 alone in the same model reduced tumor burden by ∼60% [15]. The enhanced efficacy observed here thus suggests that in addition to BMI1, the other targets of miR-15a and miR-16 are pathologically relevant and support ovarian tumor growth.

In summary, multiple mechanisms that promote OvCa progression and chemo-resistance such as EMT, drug transport and enhanced clonal growth can be inhibited by miR-15a and miR-16. With the advent of miRNA replacement therapy some of which are in Phase 2 clinical trials [20], miR-15a and miR-16 represent novel ammunition in the anti-OvCa arsenal.

## MATERIALS AND METHODS

### Cell culture

The human OvCa cell lines A2780 and A2780-CP20 (developed by sequential exposure of the A2780 parental cell line to increasing concentrations of cisplatin, henceforth termed CP20 was a kind gift from Dr. Anil Sood, MD Anderson Cancer Center) [24], OVSAHO (Japanese Collection of Research Bioresources Cell Bank), and OVCAR4 (kind gift from Dr. Ronny I. Drapkin, formerly at Dana-Farber Cancer Institute, Boston, MA, USA) were cultured in RPMI (Corning, Manassas, VA, USA) supplemented with 10% heat inactivated FBS (Gibco, Grand Island, NY, USA) and 100 units penicillin & 100 μg streptomycin/ml (Invitrogen, Rockford, IL, USA) in 5% CO_2_ humidified atmosphere. Ovarian epithelial cell line (OSE tsT/hTERT, henceforth OSE) (kind gift from Dr. V. Shridhar, Mayo Clinic, Rochester, MN) [46] was cultured in a 1:1 Media 199:MCDB 105 with 100 units penicillin & 100 μg streptomycin/ml and 15% heat-inactivated FBS.

### Patients and clinicopathologic evaluation

Publicly available Level3 Illumina RNASeqv2 and miRNASeq data for ovarian serous cystadenocarcinoma patients from TCGA was downloaded (http://tcga-data.nci.nih.gov/). The clinical information was retrieved through the cBioPortal (http://www.cbioportal.org/). Statistical analysis was performed in R (version 3.0.1) (http:///www.r-project.org/) and statistical significance was defined as a p-value < 0.05.

To evaluate correlation between BMI1/miR-15a miR-16 expression with the patient overall survival, patients were grouped into percentiles according to mRNA/miRNA expression. The Log-rank test was employed to determine the association between mRNA/miRNA expression and overall survival. Cut-off points to significantly split (log-rank test p-value <0.05) the samples into low/high mRNA/miRNA groups were recorded. The cut-off to optimally separate the patients in high/low (min p-value) group was 0.63 for BMI1 and 0.41 for miR-16. The Kaplan-Meier method was used to generate survival curves [47]. We then considered whether adding second expression level added information. A fixed cut-off for BMI together with a fixed cut-off for miR-16 splits the cohort in four groups corresponding to low/high BMI1 and low/high miR-16 expression. For each pair of cut-offs we contrasted the two groups linked to a negative association: tumors with high levels of BMI1 and low levels of miR-16 versus tumors with low levels of BMI1 and high levels of miR-16. We recorded the best separation obtained (min p-value) for the pair and noticed that the difference in median survival time between the two groups contrasted is significantly larger than the difference between the groups classified into high/low based on the expression of BMI1 or miR-16 alone. We also generated survival curves for the 3 groups (the two groups linked to a negative association and the rest of the cases) and verified the largest difference in survival time between the two groups contrasted.

### RNA isolation, reverse transcription and quantitation of miR expression levels

For quantification of endogenous or transfected miR-15a and miR-16 expression levels total RNA was isolated using TRI Pure reagent (Roche, Indianapolis, IN 46250-0414 USA), quantified, 1 μg RNA was reverse transcribed and quantitative polymerase chain reaction was performed using TaqMan® miRNA assays (Cat No. 000389 for hsa-miR-15a, Cat No. 000391 for hsa-miR-16, and Cat. No 001973 for U6; from Life Technologies, Grand Island, NY, US). Relative miR expression levels were calculated using the comparative C_t_ method with U6 as the normalizer [6].

### miR transfection

miR transfection was performed using Lipofectamine® RNAiMAX (Invitrogen, Grand Island, NY, USA) and miRIDIAN Mimic Negative Control (Dharmacon, cat No-CN-001000-01, Fisher Scientific, Pittsburgh PA, 15275, USA) or Pre-miR precursor hsa-miR-15a or hsa-miR-16 (Ambion®, Grand Island, NY 14072 USA). For the BMI1 rescue experiments, CP20 cells were transfected with 200 nM miR along with 500 ng of pcDNA3-Flag-BMI1 (kind gift from Damu Tang, McMaster University, Hamilton, ON, Canada) [48] or empty vector construct using Lipofectamine 2000 (Invitrogen, Grand Island, NY 14072 USA).

### Cell lysis and western blotting

Cellular lysates were prepared in RIPA (Boston Bioproducts, Ashland, MA, USA) containing Halt™ Protease and Phosphatase Inhibitor Cocktail (Thermo Scientific, Grand Island, NY 14072 USA). Protein was quantified using the BCA assay kit (Thermo Scientific, Grand Island, NY 14072), separated on SDS-PAGE gels and transferred to the Immun-Blot® PVDF membrane (Bio-Rad, Hercules, California 94547USA). Membranes were blocked in 5% nonfat milk and incubated with the following primary antibodies; BMI1 (Invitrogen at 1:1000 dilution), Fibronectin, N-Cad, E-Cad (BD Biosciences at 1:1000) EMA (Dako at 1:500 dilution), EPCAM, Snail, (Cell signaling technology at 1:1000 dilution), ATP7B (Protein Tech at 1:500) TWIST1, α tubulin and β actin (Sigma, TWIST1 at 1:1000, α tubulin and β actin at 1:10,000 dilutions), Secondary antibodies (from Sigma) were used at a concentration of 1:10,000.

### Cell proliferation assay

The CyQUANT® NF Cell Proliferation Assay (Invitrogen Grand Island, NY 14072 USA) was used according to the manufacturer's protocol. Briefly, 24h post miR transfection, 5000 cells were plated in a clear bottom black-well plate and fluorescence intensity at 485 nm excitation and 530 nm emissions was measured after another 24h, 48h and 72h using the CLARIOstar (BMG Labtech, Ortenberg, Germany). Values were normalized with fluorescence levels at 24h and plotted as fold change relative to 24h. Similarly transfected cells were treated with cisplatin as indicated for 48h. Percent cell viability was evaluated by comparing with respective vehicle treated cells.

### Soft agar assay

48h post miR transfection 1×10^3^ CP20 or OVSAHO cells in RPMI medium containing 10% FBS with 0.3% agar (SeaPlaque™ GTG™ Agarose, Lonza Allendale, NJ 0740,1USA) were seeded on top of 0.6% agar in the same medium in each well of 12-well plate. After 10 (CP20) or 14 (OVSAHO) days, the colonies were stained with 0.1% crystal violet, imaged using Leica EZ4HD (Buffalo Grove, IL 60089 USA). 9 images from 3 independent experiments were quantified using Image J (image processing and analysis in Java, NIH) in a blinded fashion.

### Intracellular cisplatin measurement

Uptake of cisplatin was determined by ICP-MS. 24h post miR CTL or miR-15a + miR-16 transfection CP20 cells were treated with cisplatin for 48h, washed with ice cold PBS, scraped and pellet was used for intracellular platinum quantitation. Briefly the cell pellets were digested for 1h in Optima grade concentrated nitric acid (HNO_3_, 150 μL) in a hot block digester set at 105°C, followed by addition of Optima grade hydrochloric acid (HCl, 15 μL) and continued heating for another 30min. The sample solution was then diluted to 5 mL with ultrapure water. The sample solution was then analyzed for Pt using a VG Axiom high-resolution ICP-MS. Cisplatin uptake experiments were repeated 2 times each from pooled samples of three independent experiments [32].

### Gelatin degradation assay

Acid-washed coverslips were first coated with 50 ug/mL poly-L-lysine for 20min at room temperature, and then fixed with 0.5% glutaraldehyde for 15min. Gelatin matrix was prepared by mixing 0.2% gelatin and Oregon Green® 488 Gelatin Conjugate (Life Technologies, Rockford, IL, USA) at an 8:1 ratio. After coating for 10min, coverslips were washed with PBS and quenched with 5 mg/ml sodium borohydride for 15min followed by washing with PBS. For degradation assay, 20,000 cells were seeded in each well of a 24-well plate containing acid washed cover slips. 48h after cell seeding, cells were fixed in 4% Paraformaldehyde (PFA) and stained with Alexa Fluor® 555 Phalloidin (Life Technologies, Rockford, IL, USA) for 15min at room temperature. The cells were washed with PBS and mounted with VECTASHIELD® mounting medium containing DAPI (Vector Laboratories) [35]. Images were acquired at 40X (with 1.6X Optovar) using the Zeiss Axio-Observer Z1 (Göttingen, Germany). Cells that degraded the ECM at focal adhesions (FA) sites were scored as positive, and more than 100 random cells from three independent experiments were quantified. The percentage of cells showing degradation was plotted and statistical significance was analyzed by two-tailed *t* test, P<0.05 was considered significant.

### Preparation of miR-incorporated liposome (siRNA/DOPC) nanoparticles

In order to deliver miR-15a and miR-16 into tumor, we first incorporated these miRNA mimics (mirVana® miRNA mimic: Cat No. MC10235 for hsa-miR-15a-5p and Cat No. MC10339 for hsa-miR-16-5p (Life Technologies, Rockford, IL, USA), into neutral liposome DOPC(1,2-dioleoyl-sn-glycero-3-phosphatidylcholine). Liposome nanoparticles were prepared as described earlier [1]. Briefly DOPC and miR were mixed in the presence of excess tertiary butanol at a ratio of 1:10 (w/w) miR:DOPC. TWEEN 20 was added to the mixture in a ratio of 1:19 TWEEN 20:miR/DOPC. The mixture was vortexed, frozen in an acetone and dry ice bath, and lyophilized. Before *in vivo* administration, the preparation was hydrated with phosphate-buffered saline at ambient temperature.

### *In-vivo* model and tissue processing

Female athymic nude mice were purchased from the National Cancer Institute, Frederick Cancer Research and Development Center (Frederick, MD) and maintained according to guidelines set forth by the American Association for Accreditation of Laboratory Animal Care and the US Public Health Service policy on Human Care and Use of Laboratory Animals.

Before injection, platinum-resistant OvCa CP20 cells were washed twice with PBS, trypsinized, centrifuged and re-suspended in HBSS (Invitrogen, Grand Island, NY 14072 USA). Cell viability was confirmed by trypan blue exclusion. Tumors were established by i.p. injection of 1 × 10^6^ cells. Once established, this tumor model reflects the growth pattern of advanced OvCa [15, 49].

To assess the effects of miR therapy alone and in combination with cisplatin on tumor growth, treatment with miR (150 mg/kg i.p. twice weekly) and cisplatin (160 mg/mouse once a week) was initiated one week after injection of tumor cells. Mice were divided into eight groups (n = 10 mice per group): (*a*) Control miR + DOPC, (*b*) miR-15a + DOPC, (*c*) miR-16 + DOPC, (d) control miR/DOPC + cisplatin, (*e*) miR-15a/DOPC + Cisplatin, (*f*) miR-16/DOPC + Cisplatin (g) Combo (miR-15a + miR-16/DOPC), and (h) Combo (miR-15a + miR-16/DOPC) + Cisplatin. Treatment was continued until any mice became moribund (typically 4 weeks following tumor cell injection). At the time of sacrifice, mouse weight, tumor weight, number of nodules, and distribution of tumors were recorded. Tissue samples were snap frozen for lysate preparation or fixed in formalin for paraffin embedding. The individuals who did the necropsies, tumor collections, and tissue processing were blinded to the treatment group assignments.

### Immunohistochemistry

Formalin-fixed, paraffin-embedded tissue sections were deparaffinized in xylene, rehydrated in graded alcohol, and transferred to PBS. After antigen retrieval with Diva Decloaker solution (Biocare Medical, CA 94520, USA), the endogenous peroxidase was blocked with 3% hydrogen peroxide in methanol for 15min. After washing with PBS, sections were incubated with protein block (5% normal horse serum and 1% normal goat serum) for 20min at ambient temperature, followed by incubation with anti-Ki67 (BioCare Medical, Concord, CA, USA) or BMI1 (EMD Millipore Temecula, California 92590, USA) antibodies overnight at 4°C. After washing with PBS, Ki67 sections were incubated with horseradish peroxidase-conjugated goat anti-rabbit (Serotec, Harlan Bioproducts for Science) for 1h at ambient temperature. BMI1 sections were incubated with biotinylated goat anti-mouse IgG (Biocare Medical, Concord, CA, USA) for 25min followed by washing and incubating with HRP-label streptavidin for 20min. Slides were stained with DAB substrate (Research Genetics), washed, and counterstained with Gil No.3 hematoxylin (BioGenex Laboratories) for 20sec. To evaluate the cell proliferation, the proliferative indices were determined by counting the number of Ki67-positive cells of the total number of cells in five randomly selected high-power fields exclusive of necrotic areas at 100x magnification [15]. The data were expressed as the percentage of Ki67-positive cells. Two metrics were used for BMI1 evaluation: percentage of area stained (both nuclear and cytosolic) and number of positive cells. All staining was quantified by two investigators in a blinded fashion.

### Statistical analysis

All the experiments were performed in triplicate and repeated independently three times. Transfection experiments were performed by pooling cells from two different independent transfections, which were again performed in duplicate. Data are expressed as means ± standard deviation (SD). Student's *t* test was used for statistical analysis with significance set at P < 0.05.

For animal experiments, 10 mice were assigned per treatment group. This sample size gave 80% power to detect a 50% reduction in tumor weight with 95% confidence [50]. Mouse and tumor weights for each group were compared using Student's *t* test (for comparisons of two groups). Statistical analyses were done using Statistical Package for the Social Sciences 12.0 for Windows (SPSS, Inc.). A two-tailed P≤0.05 was deemed statistically significant.

## SUPPLEMENTARY FIGURE



## References

[1] Yang D, Sun Y, Hu L, Zheng H, Ji P, Pecot CV, Zhao Y, Reynolds S, Cheng H, Rupaimoole R, Cogdell D, Nykter M, Broaddus R (2013). Integrated analyses identify a master microRNA regulatory network for the mesenchymal subtype in serous ovarian cancer. Cancer cell.

[2] Bendoraite A, Knouf EC, Garg KS, Parkin RK, Kroh EM, O'Briant KC, Ventura AP, Godwin AK, Karlan BY, Drescher CW, Urban N, Knudsen BS, Tewari M (2010). Regulation of miR-200 family microRNAs and ZEB transcription factors in ovarian cancer: evidence supporting a mesothelial-to-epithelial transition. Gynecologic oncology.

[3] Parikh A, Lee C, Joseph P, Marchini S, Baccarini A, Kolev V, Romualdi C, Fruscio R, Shah H, Wang F, Mullokandov G, Fishman D, D'Incalci M (2014). microRNA-181a has a critical role in ovarian cancer progression through the regulation of the epithelial-mesenchymal transition. Nat Commun.

[4] Sorrentino A, Liu CG, Addario A, Peschle C, Scambia G, Ferlini C (2008). Role of microRNAs in drug-resistant ovarian cancer cells. Gynecologic oncology.

[5] van Jaarsveld MT, Helleman J, Berns EM, Wiemer EA (2010). MicroRNAs in ovarian cancer biology and therapy resistance. The international journal of biochemistry & cell biology.

[6] Bhattacharya R, Nicoloso M, Arvizo R, Wang E, Cortez A, Rossi S, Calin GA, Mukherjee P (2009). MiR-15a and MiR-16 control Bmi-1 expression in ovarian cancer. Cancer Res.

[7] Zhang L, Huang J, Yang N, Greshock J, Megraw MS, Giannakakis A, Liang S, Naylor TL, Barchetti A, Ward MR, Yao G, Medina A, O'Brien-Jenkins A (2006). microRNAs exhibit high frequency genomic alterations in human cancer. Proceedings of the National Academy of Sciences of the United States of America.

[8] Calin GA, Dumitru CD, Shimizu M, Bichi R, Zupo S, Noch E, Aldler H, Rattan S, Keating M, Rai K, Rassenti L, Kipps T, Negrini M (2002). Frequent deletions and down-regulation of micro-RNA genes miR15 and miR16 at 13q14 in chronic lymphocytic leukemia. Proceedings of the National Academy of Sciences of the United States of America.

[9] Marcus CS, Maxwell GL, Darcy KM, Hamilton CA, McGuire WP (2014). Current approaches and challenges in managing and monitoring treatment response in ovarian cancer. Journal of Cancer.

[10] Ozols RF, Bundy BN, Greer BE, Fowler JM, Clarke-Pearson D, Burger RA, Mannel RS, DeGeest K, Hartenbach EM, Baergen R, Gynecologic Oncology G (2003). Phase III trial of carboplatin and paclitaxel compared with cisplatin and paclitaxel in patients with optimally resected stage III ovarian cancer: a Gynecologic Oncology Group study. Journal of clinical oncology.

[11] Matsuo K, Lin YG, Roman LD, Sood AK (2010). Overcoming platinum resistance in ovarian carcinoma. Expert opinion on investigational drugs.

[12] Wang H, Wang L, Erdjument-Bromage H, Vidal M, Tempst P, Jones RS, Zhang Y (2004). Role of histone H2A ubiquitination in Polycomb silencing. Nature.

[13] Wang J, Ma Y, Cooper MK (2013). Cancer stem cells in glioma: challenges and opportunities. Translational cancer research.

[14] Kreso A, van Galen P, Pedley NM, Lima-Fernandes E, Frelin C, Davis T, Cao L, Baiazitov R, Du W, Sydorenko N, Moon YC, Gibson L, Wang Y (2014). Self-renewal as a therapeutic target in human colorectal cancer. Nature medicine.

[15] Wang E, Bhattacharyya S, Szabolcs A, Rodriguez-Aguayo C, Jennings NB, Lopez-Berestein G, Mukherjee P, Sood AK, Bhattacharya R (2011). Enhancing chemotherapy response with Bmi-1 silencing in ovarian cancer. PloS one.

[16] Lukacs RU, Memarzadeh S, Wu H, Witte ON (2010). Bmi-1 is a crucial regulator of prostate stem cell self-renewal and malignant transformation. Cell stem cell.

[17] Meng X, Wang Y, Zheng X, Liu C, Su B, Nie H, Zhao B, Zhao X, Yang H (2012). shRNA-mediated knockdown of Bmi-1 inhibit lung adenocarcinoma cell migration and metastasis. Lung cancer.

[18] Wang Y, Zhe H, Ding Z, Gao P, Zhang N, Li G (2012). Cancer stem cell marker Bmi-1 expression is associated with basal-like phenotype and poor survival in breast cancer. World journal of surgery.

[19] Cao L, Bombard J, Cintron K, Sheedy J, Weetall ML, Davis TW (2011). BMI1 as a novel target for drug discovery in cancer. Journal of cellular biochemistry.

[20] Lammers P BA (2011). The Therapeutic Potential of microRNAs. Innovations in Pharmaceutical Technology.

[21] Yang GF, He WP, Cai MY, He LR, Luo JH, Deng HX, Guan XY, Zeng MS, Zeng YX, Xie D (2010). Intensive expression of Bmi-1 is a new independent predictor of poor outcome in patients with ovarian carcinoma. BMC Cancer.

[22] Zhang F, Sui L, Xin T (2008). Correlations of BMI-1 expression and telomerase activity in ovarian cancer tissues. Exp Oncol.

[23] Teshima K, Nara M, Watanabe A, Ito M, Ikeda S, Hatano Y, Oshima K, Seto M, Sawada K, Tagawa H (2014). Dysregulation of BMI1 and microRNA-16 collaborate to enhance an anti-apoptotic potential in the side population of refractory mantle cell lymphoma. Oncogene.

[24] Sood AK, Seftor EA, Fletcher MS, Gardner LM, Heidger PM, Buller RE, Seftor RE, Hendrix MJ (2001). Molecular determinants of ovarian cancer plasticity. The American journal of pathology.

[25] Domcke S, Sinha R, Levine DA, Sander C, Schultz N (2013). Evaluating cell lines as tumour models by comparison of genomic profiles. Nature communications.

[26] Freedman VH, Shin SI (1974). Cellular tumorigenicity in nude mice: correlation with cell growth in semi-solid medium. Cell.

[27] Mangala LS, Zuzel V, Schmandt R, Leshane ES, Halder JB, Armaiz-Pena GN, Spannuth WA, Tanaka T, Shahzad MM, Lin YG, Nick AM, Danes CG, Lee JW (2009). Therapeutic Targeting of ATP7B in Ovarian Carcinoma. Clinical cancer research.

[28] Lewis BP, Burge CB, Bartel DP (2005). Conserved seed pairing, often flanked by adenosines, indicates that thousands of human genes are microRNA targets. Cell.

[29] John B, Enright AJ, Aravin A, Tuschl T, Sander C, Marks DS (2004). Human MicroRNA targets. PLoS biology.

[30] Kiriakidou M, Nelson PT, Kouranov A, Fitziev P, Bouyioukos C, Mourelatos Z, Hatzigeorgiou A (2004). A combined computational-experimental approach predicts human microRNA targets. Genes & development.

[31] Kertesz M, Iovino N, Unnerstall U, Gaul U, Segal E (2007). The role of site accessibility in microRNA target recognition. Nature genetics.

[32] Xiong X, Arvizo RR, Saha S, Robertson DJ, McMeekin S, Bhattacharya R, Mukherjee P (2014). Sensitization of ovarian cancer cells to cisplatin by gold nanoparticles. Oncotarget.

[33] Rosano L, Cianfrocca R, Spinella F, Di Castro V, Nicotra MR, Lucidi A, Ferrandina G, Natali PG, Bagnato A (2011). Acquisition of chemoresistance and EMT phenotype is linked with activation of the endothelin A receptor pathway in ovarian carcinoma cells. Clinical cancer research.

[34] Razidlo GL, Schroeder B, Chen J, Billadeau DD, McNiven MA (2014). Vav1 as a central regulator of invadopodia assembly. Current biology: CB.

[35] Artym VV, Yamada KM, Mueller SC (2009). ECM degradation assays for analyzing local cell invasion. Methods in molecular biology.

[36] Calin GA, Croce CM (2006). MicroRNA-cancer connection: the beginning of a new tale. Cancer research.

[37] Zhang L, Volinia S, Bonome T, Calin GA, Greshock J, Yang N, Liu CG, Giannakakis A, Alexiou P, Hasegawa K, Johnstone CN, Megraw MS, Adams S (2008). Genomic and epigenetic alterations deregulate microRNA expression in human epithelial ovarian cancer. Proceedings of the National Academy of Sciences of the United States of America.

[38] Komatsu M, Sumizawa T, Mutoh M, Chen ZS, Terada K, Furukawa T, Yang XL, Gao H, Miura N, Sugiyama T, Akiyama S (2000). Copper-transporting P-type adenosine triphosphatase (ATP7B) is associated with cisplatin resistance. Cancer research.

[39] Andrews PA, Howell SB (1990). Cellular pharmacology of cisplatin: perspectives on mechanisms of acquired resistance. Cancer cells.

[40] Ohbu M, Ogawa K, Konno S, Kanzaki A, Terada K, Sugiyama T, Takebayashi Y (2003). Copper-transporting P-type adenosine triphosphatase (ATP7B) is expressed in human gastric carcinoma. Cancer letters.

[41] Kanzaki A, Toi M, Neamati N, Miyashita H, Oubu M, Nakayama K, Bando H, Ogawa K, Mutoh M, Mori S, Terada K, Sugiyama T, Fukumoto M (2002). Copper-transporting P-type adenosine triphosphatase (ATP7B) is expressed in human breast carcinoma. Japanese journal of cancer research: Gann.

[42] Nakayama K, Miyazaki K, Kanzaki A, Fukumoto M, Takebayashi Y (2001). Expression and cisplatin sensitivity of copper-transporting P-type adenosine triphosphatase (ATP7B) in human solid carcinoma cell lines. Oncology reports.

[43] Polyak K, Weinberg RA (2009). Transitions between epithelial and mesenchymal states: acquisition of malignant and stem cell traits. Nature reviews Cancer.

[44] Bagnato A, Rosano L (2012). Understanding and overcoming chemoresistance in ovarian cancer: emerging role of the endothelin axis. Current oncology.

[45] Ahmed N, Abubaker K, Findlay J, Quinn M (2010). Epithelial mesenchymal transition and cancer stem cell-like phenotypes facilitate chemoresistance in recurrent ovarian cancer. Current cancer drug targets.

[46] Rattan R, Narita K, Chien J, Maguire JL, Shridhar R, Giri S, Shridhar V (2010). TCEAL7, a putative tumor suppressor gene, negatively regulates NF-kappaB pathway. Oncogene.

[47] Bewick V, Cheek L, Ball J (2004). Statistics review 12: survival analysis. Crit Care.

[48] Fan C, He L, Kapoor A, Gillis A, Rybak AP, Cutz JC, Tang D (2008). Bmi1 promotes prostate tumorigenesis via inhibiting p16(INK4A) and p14(ARF) expression. Biochimica et biophysica acta.

[49] Merritt WM, Lin YG, Spannuth WA, Fletcher MS, Kamat AA, Han LY, Landen CN, Jennings N, De Geest K, Langley RR, Villares G, Sanguino A, Lutgendorf SK (2008). Effect of interleukin-8 gene silencing with liposome-encapsulated small interfering RNA on ovarian cancer cell growth. Journal of the National Cancer Institute.

[50] Nick AM, Stone RL, Armaiz-Pena G, Ozpolat B, Tekedereli I, Graybill WS, Landen CN, Villares G, Vivas-Mejia P, Bottsford-Miller J, Kim HS, Lee JS, Kim SM (2011). Silencing of p130cas in ovarian carcinoma: a novel mechanism for tumor cell death. Journal of the National Cancer Institute.

