# Supplementary material for: Therapeutic evaluation of microRNA-15a and microRNA-16 in ovarian cancer
Source: Oncotarget. 2016 Feb 23;7(12):15093–104. doi: 10.18632/oncotarget.7618 (PMC4924772; doi:10.18632/oncotarget.7618)
Supplement: Supplementary file 1 [file oncotarget-07-15093-s001.pdf]

SUPPLEMENTARY FIGURE

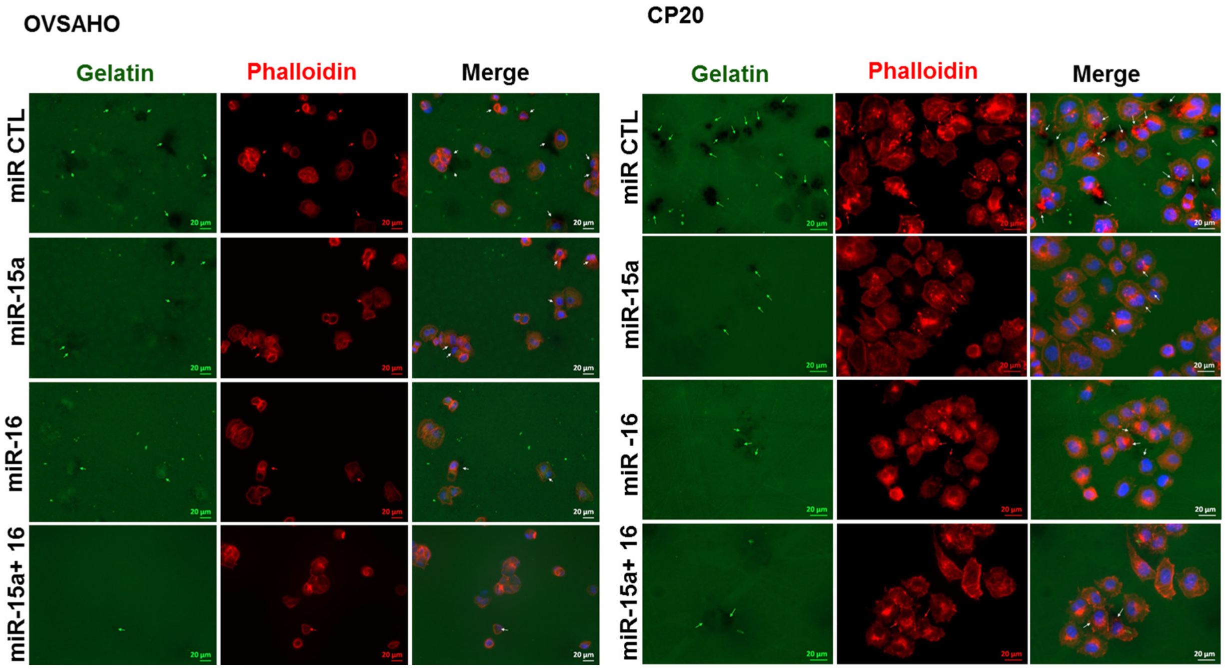

Supplementary Figure S1: Effect of miR-15a and miR-16 on ECM degradation. miR CTL, miR-15a, miR-16 or miR-15a+ miR-16 transfected OVSAHO and CP20 cells were plated on Oregon Green® 488 Gelatin coated coverslips for 48h, fixed, stained with Alexa Fluor® 555 Phalloidin, mounted in Vecatshield containing DAPI and images acquired. Scale bar represent 20µm. Arrows indicate areas of gelatin degradation.
